# Survival outcomes in an older US population with advanced melanoma and central nervous system metastases: SEER‐Medicare analysis

**DOI:** 10.1002/cam4.3256

**Published:** 2020-07-15

**Authors:** Natalia Sadetsky, Alexandra Hernandez, Chris J. Wallick, Edward F. McKenna, Andy Surinach, Dawn E. Colburn

**Affiliations:** ^1^ Real World Data Science – Product Development Genentech, Inc South San Francisco CA USA; ^2^ US Medical Affairs Genentech, Inc South San Francisco CA USA; ^3^ Genesis Research LLC Hoboken NJ USA; ^4^ Product Development – Oncology Genentech, Inc South San Francisco CA USA

**Keywords:** CNS metastases, melanoma, overall survival, radiotherapy, systemic treatment

## Abstract

**Background:**

Central nervous system (CNS) metastasis is common in advanced melanoma patients. New treatment options have improved overall prognosis, but information is lacking for patients with CNS metastases. We investigated treatment patterns and survival outcomes in older melanoma patients with and without CNS metastases.

**Methods:**

A retrospective analysis of SEER‐Medicare, a population‐based linked database, was undertaken in patients aged > 65 years with advanced melanoma diagnosed from 2004 to 2011 and followed until 2013.

**Results:**

A total of 2522 patients were included. CNS metastases were present in 24.8% of patients at initial metastatic diagnosis; 16.5% developed CNS metastases during follow‐up. Chemotherapy was the most common treatment regardless of CNS metastases. Overall survival (OS) was better for patients without CNS metastases (median, 9.5 months; 95% confidence interval [CI], 8.8‐10.2) vs patients with CNS metastases (3.63 months; 95% CI, 3.4‐3.9). Among patients with CNS metastases, median OS for targeted therapy, immunotherapy, and chemotherapy was 6 (95% CI, 2.5‐9.6), 5.5 (95% CI, 3.8‐7.5), and 4.5 (95% CI, 3.8‐5.4) months, respectively, vs 2.4 (95% CI, 2.1‐2.7) and 2.1 (95% CI, 1.8‐2.7) months for local radiotherapy and no treatment, respectively. Stereotactic radiosurgery demonstrated higher OS vs whole‐brain radiation therapy (median, 4.98 [95% CI, 3.5‐7.5] vs 2.4 [95% CI, 2.1‐2.7] months).

**Conclusion:**

Patients with CNS metastases from melanoma remain a population with high unmet medical need despite recent advances in treatment. Systemic treatments (eg, BRAF‐targeted therapy and immunotherapy) and stereotactic radiosurgery demonstrated meaningful but modest improvements in OS. Further explorations of combinations of radiotherapy, BRAF‐targeted therapies, and immunotherapies are needed.

## Graphical abstract

This retrospective, population‐based analysis of older patients with advanced melanoma demonstrated that patients with central nervous system (CNS) metastases have limited treatment options and poor survival outcomes. Improved survival was observed for patients with CNS metastases treated with BRAF‐targeted therapy or immunotherapy, but significant unmet medical need remains.

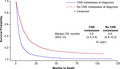

## 1 INTRODUCTION

Melanoma is one of the least common forms of skin cancer, but when advanced it is the most likely to be fatal.^1^ Among solid tumors, melanoma has the highest risk of spreading to the central nervous system (CNS), with CNS metastases present in approximately 20% of patients at diagnosis; up to 50% of patients with melanoma develop CNS metastases over the course of disease.^2^ Compared with other malignancies (eg, breast, lung) in which CNS metastases frequently occur, patients with melanoma have an increased risk of death due to CNS metastases.^3^ CNS involvement in patients with metastatic melanoma is associated with poor prognosis (median survival, 4‐6 months), and failure to control CNS metastases is a leading cause of death.^2^, ^4^ Standard treatments for patients with melanoma with CNS metastases include whole‐brain radiation therapy (WBRT) and stereotactic radiosurgery (SRS), alone or in combination, and cytotoxic chemotherapy; however, none of these treatments has a significant impact on overall survival (OS).^5^

Over the past decade, considerable advances have been made in the treatment of metastatic melanoma, with 10 new regimens approved between 2011 and 2015.^2^ Although therapies targeting *BRAF* mutations and immunotherapies have demonstrated significant improvements in progression‐free survival and OS, a majority of pivotal phase three trials excluded patients with CNS metastases.^6^, ^7^, ^8^, ^9^, ^10^ These new treatment options might increase the probability of development and diagnosis of CNS metastases simply because of extended survival; alternatively, the incidence may decrease due to the blood‐brain barrier–penetrating attributes of several new therapies.^2^ The presence of CNS metastases is a key determinant of patient prognosis, so understanding treatment patterns and survival outcomes in real‐world populations could better inform treatment decisions. Although several studies evaluating targeted therapies (BRAF and/or MEK inhibitors) and immunotherapies (anti–programmed cell death protein 1 [PD‐1] and/or anti‐cytotoxic T‐lymphocyte–associated protein 4 [CTLA‐4] therapies) have recently demonstrated clinical activity in patients with melanoma with CNS metastases,^11^, ^12^, ^13^, ^14^ information about elderly patients remains limited.

The primary objective of this study was to evaluate OS in patients with advanced melanoma with and without CNS metastases in a population‐based US cohort of older adults (aged > 65 years). A secondary objective was to describe treatment modalities among patients with CNS metastases.

## 2 METHODS

### 2.1 Data source

This study utilized cancer registry data from the Surveillance Epidemiology and End Results (SEER) Program maintained by the National Cancer Institute (NCI), linked to enrollment and claims data from Medicare (SEER‐Medicare). Methodology, content, and data collection for SEER‐Medicare has been described previously.^15^, ^16^ The SEER registry gathers information on cancer incidence and mortality in the United States from 20 population‐based registries and covers approximately one third of the US population. In addition, SEER collects information on patient demographics and cancer characteristics, including site, stage, tumor morphology, treatment, and survival status. Information on utilization of inpatient and outpatient services and further patterns of care is available through incorporation of Medicare claims data in the SEER‐Medicare Linked Database, which is a joint effort of NCI, SEER, and Centers for Medicare and Medicaid Services. The linking of individual identifiers between SEER and Medicare is undertaken every 2 years, and approximately 95% of all persons aged ≥ 65 years who are documented in SEER are matched to Medicare records.^15^

### 2.2 Patient eligibility

Two patient groups were included: those first diagnosed with metastatic (stage IV) melanoma between 2004 and 2011 and those diagnosed with stages 0‐III melanoma between 2004 and 2011 who had evidence of treatment for metastatic disease.

In order to have sufficient information to evaluate baseline comorbidities and prior treatments, we required that patients be enrolled in Medicare Parts A (inpatient services) and B (outpatient and physician services) and that they not have any other insurance coverage for at least 12 months before diagnosis or treatment of metastatic disease. To minimize the number of patients treated for other cancers, those with any nonmelanoma cancer diagnosis in the year before diagnosis of metastatic melanoma were excluded.

### 2.3 Timing of exposures and outcomes

We assessed exposure to therapy through records of intravenous or oral systemic therapy using International Classification of Diseases, 9th Revision, Clinical Modification (ICD‐9‐CM), Healthcare Common Procedure Coding System codes and Medicare Part D (prescription drug coverage). For confidentiality reasons, SEER provides only the calendar month of cancer diagnosis. Therefore, metastatic diagnosis date was defined as the first day of the month in which diagnosis occurred. We used date of the first Medicare claim for systemic treatment (intravenous or oral) to indicate the start of therapy. Survival was assessed using information on date of death provided by Medicare or SEER. Patients were followed for survival until December 31, 2013.

### 2.4 Patient characteristics

We describe patient clinical and demographic characteristics, including age (median and categorical), sex, race, geographical distribution, and other factors at the time of melanoma diagnosis. Using previously described methodology,^17^ we calculated Charlson comorbidity index for each patient using Medicare inpatient and outpatients claims.

Initial treatment was identified as first Medicare claim for chemotherapy (dacarbazine, cisplatin, carboplatin, or temozolomide), targeted therapy (vemurafenib, trametinib, or dabrafenib), or immunotherapy (ipilimumab) after diagnosis date. Similarly, evidence of treatment for metastatic melanoma was identified via first Medicare claim using codes for chemotherapy, targeted therapy, and immunotherapy after melanoma diagnosis.

Radiotherapy was identified using methodology described by Halaz et al.^18^ For radiotherapy, the specific anatomic site of treatment cannot be identified from Medicare claims data. Therefore, patients who underwent radiotherapy without neurological resection within 1 month before through 2 months after diagnosis of CNS metastases were included in the radiotherapy subset analysis. Time frame was selected to capture any delayed claims (due to administrative delays) and to provide sufficient time to completion of initial treatment.

A single occurrence of ICD‐9‐CM code 198.3 was used to indicate presence of metastasis to the brain or spinal cord (CNS metastasis) as recently validated in patients with lung cancer.^19^

### 2.5 Statistical analysis

Demographic and clinical characteristics for patients with and without CNS metastases were summarized separately, with frequency distributions for categorical variables and mean values with standard deviations for continuous variables. Overall and landmark survival at 6, 12, 24, and 36 months were estimated using Kaplan‐Meier methodology. The log‐rank test was used to determine significant differences between groups.

## 3 RESULTS

A total of 217 591 patients with melanoma were identified in the SEER‐Medicare database; 3146 were diagnosed with stage IV melanoma from January 2004 through December 2011, and 2018 patients were diagnosed with stages 0‐III melanoma and had evidence of treatment for metastatic disease in the same period. After applying inclusion and exclusion criteria, 2522 patients were included in the study population (Table 1). Mean age was 74.3 years and 67.6% were males. The majority of patients were white (96.1%), with black or African‐American, Asian, and Hispanic patients representing 0.9%, 0.9%, and 1.2%, respectively. The majority lived in metropolitan areas: 55.3% in big metro areas (population ≥ 1 million) and 28.5% in metro areas (population < 1 million). Smaller proportions resided in less urban and rural areas (7.7% and 2.5%, respectively). Diabetes and chronic pulmonary disease were the most common comorbidities (16.6% and 8.2%, respectively) and Charlson Comorbidity Index score was 0 in 66.5%.

**TABLE 1:** Baseline characteristics

Characteristic		All patients N = 2522		Patients with CNS metastases n = 625		Patients without CNS metastases n = 1897
Age at metastatic diagnosis, mean years (SD)		74.3 (8.8)		72.6 (8.4)		74.8 (8.8)
Age group, n (%)						
≤65 y		278 (11.0)		74 (11.8)		204 (10.8)
66‐69 y		443 (17.6)		148 (23.7)		295 (15.6)
70‐74 y		559 (22.2)		151 (24.2)		408 (21.5)
75‐79 y		538 (21.3)		125 (20.0)		413 (21.8)
80‐84 y		409 (16.2)		80 (12.8)		329 (17.3)
≥85 y		295 (11.7)		47 (7.5)		248 (13.1)
Sex, n (%)						
Male		1705 (67.6)		469 (75.0)		1236 (65.2)
Female		817 (32.4)		156 (25.0)		661 (34.8)
Race, n (%)						
White		2425 (96.2)		604 (96.6)		1820 (95.9)
Nonwhite		97 (3.8)		21 (3.4)		77 (4.1)
Marital status, n (%)						
Married		1492 (59.2)		415 (66.4)		1077 (56.8)
Unmarried		1030 (40.8)		210 (33.6)		821 (43.2)
Residence, n (%)^a^						
Big metro	1394 (55.3)		334 (53.4)		1060 (55.9)	
Metro	720 (28.5)		193 (30.9)		527 (27.8)	
Urban	149 (5.9)		33 (5.3)		116 (6.1)	
Less urban	194 (7.7)		49 (7.8)		145 (7.6)	
Rural	64 (2.5)		16 (2.7)		48 (2.5)	
CCI score, n (%)						
0		1676 (66.5)		426 (68.2)	1250 (65.9)	
1		494 (19.6)		131 (21.0)	363 (19.1)	
2		168 (6.7)		32 (5.1)	136 (7.2)	
3+		184 (7.3)		36 (5.8)	148 (7.8)	
Comorbidities, n (%)						
Chronic pulmonary disease		207 (8.2)		48 (7.7)		159 (8.4)
Diabetes		420 (16.7)		107 (17.1)		313 (16.5)
Diabetes with complications		90 (3.6)		23 (3.7)		67 (3.5)
Congestive heart failure		140 (5.6)		20 (3.2)		120 (6.3)
Cerebrovascular disease		113 (4.5)		24 (3.8)		89 (4.7)
Peripheral vascular disease		80 (3.2)		16 (2.6)		64 (3.4)
Acute myocardial infarction		26 (1.0)		NA^b^		NA^b^
Old myocardial infarction		47 (1.9)		NA^b^		NA^b^
Rheumatologic disease		47 (1.9)		NA^b^		NA^b^
Moderate/severe renal disease		122 (4.8)		24 (3.8)		98 (5.2)
Dementia		14 (0.6)		NA^b^		NA^b^
Ulcer disease		23 (0.9)		NA^b^		NA^b^
Hypothyroidism		143 (5.7)		NA^b^		NA^b^
Coagulopathy		38 (1.5)		NA^b^		NA^b^
Blood loss anemia		27 (1.1)		NA^b^		NA^b^
Deficiency anemia		70 (2.8)		18 (2.9)		52 (2.7)
Neuropathy		104 (4.1)		26 (4.2)		78 (4.1)

Abbreviations: CCI, Charlson Comorbidity Index; CNS, central nervous system; NA, not applicable; SD, standard deviation; SRS, stereotactic radiosurgery.

^a^ Big metro = counties in metropolitan areas with populations ≥ 1 million; Metro = counties in metropolitan areas with populations < 1 million; Urban = counties adjacent or nonadjacent to metropolitan areas with populations ≥ 20 000; Less urban = counties with populations of 2500 to 19 999; Rural = counties with populations < 2500.

^b^ Not reported due to occurrence in < 11 patients in either subgroup (per SEER‐Medicare regulations); the following comorbidities were also evaluated but are not presented because they occurred in <11 patients: paralysis, mild liver disease, moderate/severe liver disease, and AIDs.

### 3.1 CNS metastases

In the overall study population, 24.8% (625/2522) of patients presented with CNS metastases at diagnosis, whereas another 16.5% (417/2522) developed CNS metastases during follow‐up. Among patients diagnosed with stage IV disease, 28.1% (395/1408) presented with CNS metastases at diagnosis, with another 22.8% (230/1408) developing CNS metastases after diagnosis; mean time to development of CNS metastases after diagnosis was 12.4 months (interquartile range [IQR], 5.3‐15.5). Similar distribution was noted for patients diagnosed with stage I‐III disease, with 20.6% (230/1114) of patients presenting with CNS metastases at the time of treatment initiation and another 21.5% (187/1114) developing CNS metastases within 1 year; mean time to development of CNS metastases was 10.2 months (IQR, 4.6‐12.1).

### 3.2 Treatment exposures

Initial treatments differed based on presence or absence of CNS metastases. In general, 86.2% (539/625) of patients with CNS metastases and 66.2% (1169/1897) of patients without CNS metastases received some type of initial treatment. While less than 10% of the study population was treated with radiotherapy, most of these patients had CNS metastases at diagnosis (76.3%, 192/251). The use of chemotherapy was slightly higher among patients without CNS metastases at diagnosis (51.0%) than among those with CNS metastases (42.7%). Limited use of targeted therapy (2.9% and 2.4%) and immunotherapy (9.9% and 9.7%) was reported in patients with and without CNS metastases, respectively; however, these treatment options only became available in 2011 (Table 2).

**TABLE 2:** Initial treatment

Treatment, n (%)	All patients N = 2522	Patients with CNS metastases n = 625	Patients without CNS metastases n = 1897
BRAF‐targeted therapy	63 (2.5)	18 (2.9)	45 (2.4)
Immunotherapy	246 (9.8)	62 (9.9)	184 (9.7)
Chemotherapy	1234 (48.9)	267 (42.7)	967 (51.0)
Radiotherapy^a^	251 (10.0)	192 (30.7)	59 (3.1)
No treatment	728 (28.9)	86 (13.8)	642 (33.8)

Abbreviation: CNS, central nervous system.

^a^ Detailed information on radiotherapy is presented in Table 3.

### 3.3 Radiotherapy

Radiotherapy was utilized as initial treatment (alone or in conjunction with surgery) or as a part of treatment regimen in combination with systemic therapy (Table 3). Among patients with CNS metastases at diagnosis, radiotherapy was utilized in 30.7% (192/625). Most of these patients (80.7%, 155/192) underwent radiotherapy alone or in combination with neurosurgical resection. Fewer patients (19.7%, 37/192) received SRS in addition to neurosurgical resection. For patients without CNS metastases at diagnosis, 3.1% utilized radiotherapy during the observation period.

**TABLE 3:** Radiotherapy exposure

Treatment, n (%)	All patients N = 2522	Patients with CNS metastases n = 625	Patients without CNS metastases n = 1897
Radiotherapy—all	685 (27.2)	419 (67.1)	265 (14.1)
Monotherapy	252	192 (30.7)	59 (3.1)
Radiotherapy (±neurosurgical resection)		155 (80.7)	36 (61.0)
SRS (±neurosurgical resection)		37 (19.3)	23 (39)
Combination therapy	434	227 (36.3)	206 (10.8)
Radiotherapy (±neurosurgical resection)		139 (61.2)	130 (63.1)
SRS (±neurosurgical resection)		88 (39.8)	76 (36.9)

Abbreviations: CNS, central nervous system; SRS, stereotactic radiosurgery.

More than half (63%, 434/685) of the patients exposed to radiotherapy received it in combination with systemic therapy. Utilization of radiotherapy among these patients differed based on presence of CNS metastases, with higher use among patients with CNS metastases (36.3%) vs those without CNS metastases (10.8%) (Table 3).

### 3.4 Overall survival

**FIGURE 1:**
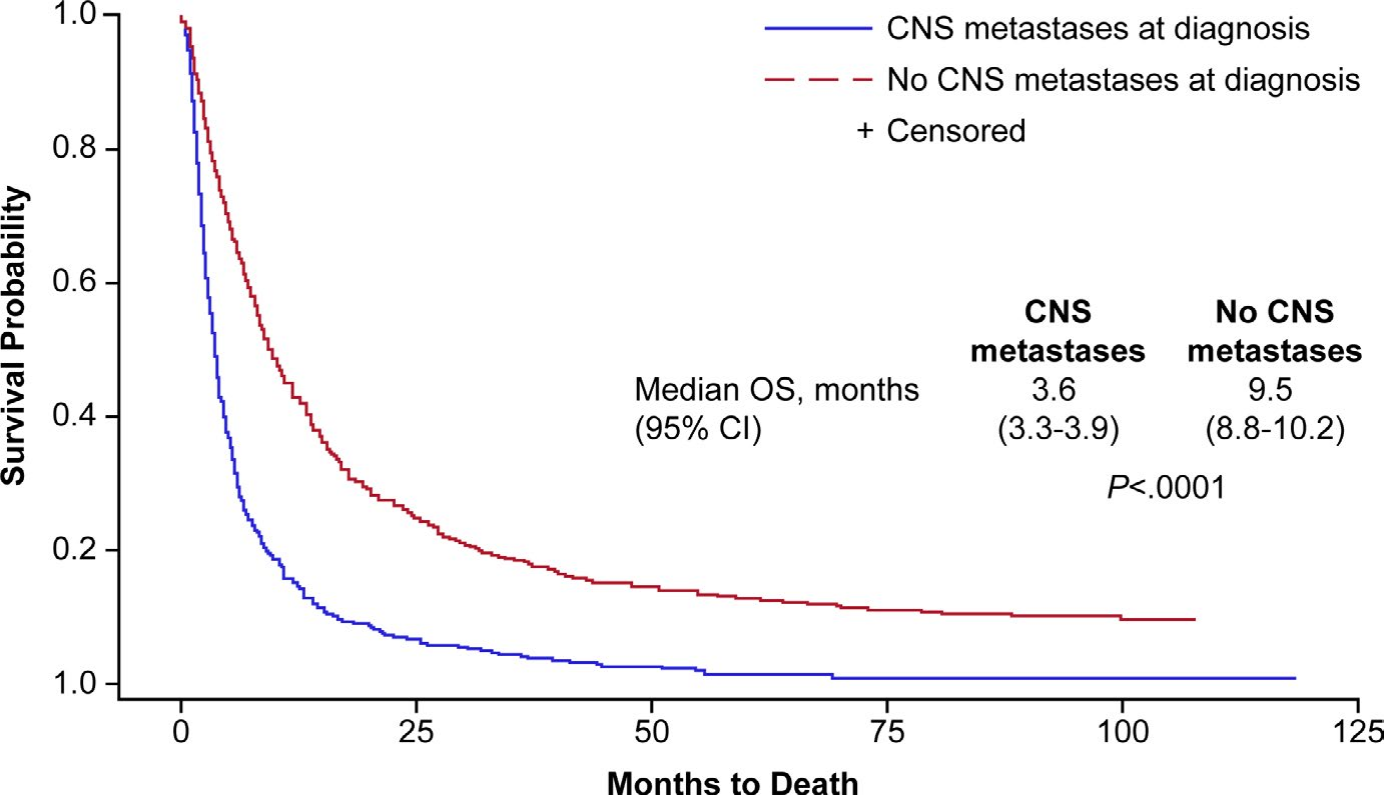
Kaplan‐Meier curves of overall survival probability in patients with and without CNS metastases at diagnosis in the entire study cohort. CI, confidence interval; CNS, central nervous system; OS, overall survival

Overall survival differed significantly based on presence or absence of CNS metastases (Figure 1). Median OS for patients with CNS metastases (3.6 months; 95% CI, 3.4‐3.9) was significantly lower than for patients without CNS metastases (9.5 months; 95% CI, 8.8‐10.2).

Among patients with CNS metastases, 30.5% (95% CI, 26.9‐34.1) were alive at 6 months, compared with 64.6% (95% CI, 62.4‐66.8) of patients without CNS metastases. Differences in OS rates continued to be noted between patients with and without CNS metastases at 12, 24, and 36 months (Table 4).

**TABLE 4:** Overall survival among patients with and without CNS metastases

Outcome	Patients with CNS metastases	Patients without CNS metastases	*P* value
Median overall survival, months (95% CI)	3.6 (3.3‐3.9)	9.5 (8.8‐10.2)	<.0001
Landmark overall survival, % (95% CI)			
6 mo	30.5 (26.9‐34.1)	64.6 (62.4‐66.8)	
12 mo	15.3 (12.4‐18.2)	43.3 (41.0‐45.6)	
24 mo	7.0 (5.0‐9.1)	25.9 (23.9‐28.0)	
36 mo	4.0 (2.6‐6.0)	18.6 (16.7‐20.5)	

Abbreviations: CI, confidence interval; CNS, central nervous system.

### 3.5 Association of treatment and survival

Among patients with CNS metastases, patients who received any treatment had better OS than those who received no treatment. Both immunotherapy‐treated and targeted therapy‐treated groups demonstrated better OS (median 5.9 months [95% CI, 2.5‐9.6] and 5.5 months [95% CI, 3.8‐7.5], respectively) compared with those treated with chemotherapy and radiotherapy (median 4.5 months [95% CI, 3.8‐5.4] and 2.7 months [95% CI, 2.4‐3.3], respectively). A similar trend was demonstrated for patients without CNS metastases (Table 5).

**TABLE 5:** Overall survival by initial treatment

Outcome	Patients with CNS metastases	Patients without CNS metastases
Median overall survival, months (95% CI)		
Targeted therapy	5.9 (2.5‐9.6)	9.5 (4.8‐13.8)
Immunotherapy	5.5 (3.8‐7.5)	17.1 (13.3‐22.4)
Chemotherapy	4.5 (3.8‐5.4)	11.2 (10.4‐12.7)
Radiotherapy	2.7 (2.4‐3.3)	NA
None	2.1 (1.8‐2.7)	6.2 (5.3‐7.2)

Abbreviations: CI, confidence interval; CNS, central nervous system; NA, not applicable.

**FIGURE 2:**
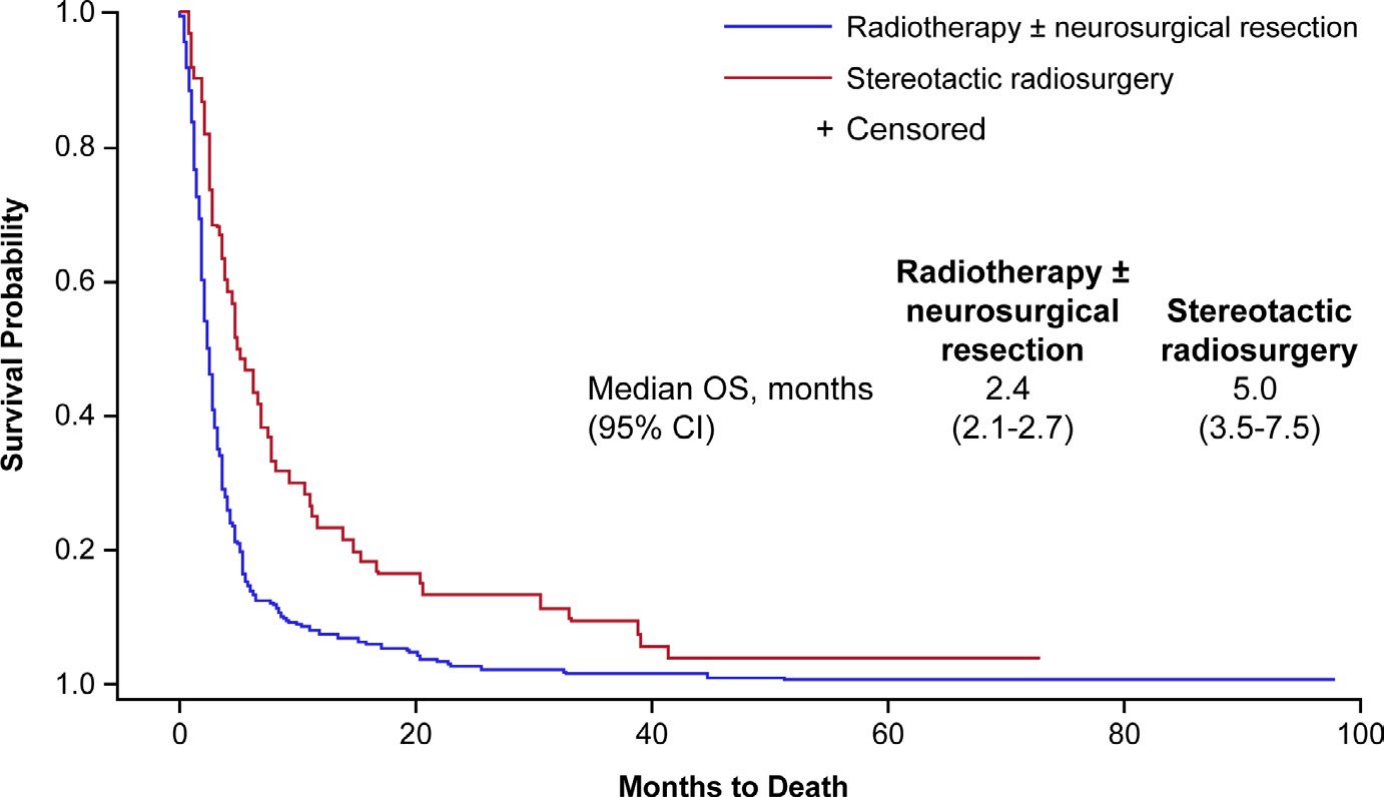
Kaplan‐Meier curves of overall survival probability by type of radiotherapy (radiotherapy ± neurosurgical resection vs stereotactic radiosurgery) among patients treated with radiotherapy. CI, confidence interval; OS, overall survival

Among patients treated with radiotherapy, those who received SRS had a median OS of 5.0 months (95% CI, 3.5‐7.5) compared with 2.4 months (95% CI, 2.1‐2.7) for those who received conventional radiotherapy (Figure 2).

## 4 DISCUSSION

Among an older population of patients with advanced melanoma in SEER‐Medicare, CNS metastases contributed significantly to overall disease burden and were associated with poor survival outcomes. CNS metastases were prevalent at initial metastatic diagnosis, with 24.8% displaying evidence of brain involvement. Additionally, 16.5% of patients who did not have CNS metastases at diagnosis or at the time of treatment developed CNS metastases within 1 year. Our results are consistent with current published literature. In a randomized controlled study evaluating the impact of biochemotherapy on developing CNS metastases in patients with metastatic melanoma, observed cumulative CNS failure rates at 1 year were 31.1% and 20.6% with dacarbazine‐ and temozolomide‐based regimens, respectively.^20^ Similar to previous studies, we found that presence of CNS metastasis was associated with significantly lower survival compared with patients without CNS metastases, underscoring the continued high unmet medical need for this population.

Management of patients with CNS metastases continues to be challenging. Survival outcomes with conventional radiotherapy were similar to those observed for patients who did not receive any tumor‐directed treatment. This finding is consistent with results of a large randomized study (QUARTZ) of patients with lung cancer with CNS metastases that demonstrated no benefit in survival or improved quality of life for patients receiving WBRT versus best supportive care.^21^ In our analysis, SRS was associated with significantly improved survival vs WBRT in this unselected population, although absolute improvement was modest. It should be noted that treatment benefit of SRS is optimized when used in a selected subset of patients based on clinical algorithms (ie, recursive partitioning analysis [RPA] score or Graded Prognostic Assessment [GPA]).^22^ The role of SRS in the management of CNS metastases from melanoma, particularly in combination with newer systemic treatments, remains to be defined.

Historically, systemic treatment (ie, chemotherapy) had limited clinical activity against metastatic melanoma, including disease within the CNS. In our study, patients with melanoma with CNS metastases who received systemic treatment had improved survival outcomes relative to patients who received local treatment only or no tumor‐directed therapy. Patients receiving targeted therapy or immunotherapy had numerically better outcomes than those treated with chemotherapy, local therapies, or no tumor‐directed treatment. Our findings are consistent with recent reports evaluating the clinical activity of BRAF‐targeted therapies or immune checkpoint inhibitors in patients with melanoma CNS metastases. Recent studies by Dummer et al and Long et al reported median OS of 5.3 months (95% CI, 3.9‐6.6) and 7.5 months (5.3‐not estimable), respectively, in patients with CNS metastases treated with targeted therapy.^23^, ^24^ A slightly higher OS of 8.9‐9.6 months was reported in another study by McArthur et al; however, patients in that study were significantly younger.^14^ A systematic review of survival outcomes in this patient population, including 22 studies and 2153 patients receiving targeted therapy or immunotherapy (predominantly ipilimumab), demonstrated evidence of clinical activity with median OS of 7.9 months (IQR, 7.1‐8.3) and 7.0 months (IQR, 7.0‐12.7) for targeted therapy and immunotherapy, respectively.^25^ Clinical activity has also been demonstrated with the anti–PD‐1 therapies nivolumab and pembrolizumab, with median OS of 9.9 months (95% CI, 6.9‐17.7). Patients with symptomatic disease and those requiring corticosteroids had worse OS outcomes, with median OS of 5.7 and 4.8 months, respectively.^26^

Given the clinical activity observed with newer systemic treatments, combination therapy is now being explored. Combinations of BRAF/MEK‐targeted therapy or immune checkpoint inhibitors have produced objective responses in the majority (~55%) of patients with asymptomatic CNS metastases.^11^, ^27^ Newer systemic treatments are also being combined with SRS. A recent retrospective analysis of 310 patients who received SRS in combination with anti–PD‐1, anti–CTLA‐4, BRAF inhibitors ± MEK inhibitors, or chemotherapy demonstrated improved OS in patients receiving immune checkpoint inhibitors or BRAF‐targeted therapy vs chemotherapy.^13^ Patients with symptomatic CNS disease and those with asymptomatic disease requiring corticosteroids remain an area of unmet medical need. Combinatorial strategies represent a viable and promising management option and merit further exploration in clinical studies.

Several important limitations to our study should be noted. Patients who were diagnosed with earlier stages of melanoma and had evidence of treatment for metastatic disease were identified based on treatment information collected for reimbursement purposes and may have limited sensitivity and specificity. Clinical information was limited to that available in the SEER registry. Specifically, information on performance status, number of CNS lesions, and status of extracranial disease needed for RPA or GPA scoring was not available. This absence may limit the ability to accurately assess the benefit of specialized radiotherapy techniques, such as SRS. As the SEER‐Medicare linked database is limited to patients aged > 65 years, our study population may not be generalizable to all patients with metastatic melanoma in the United States. In addition, although we had extensive information on treatment utilization for older patients with metastatic melanoma, we were only able to assess those who were followed up to 2013. Thus, most current treatments were not captured.

This study has several important strengths. With a rapidly changing treatment landscape, the availability of Medicare Part D data allowed us to evaluate emerging oral therapies for metastatic melanoma. By utilizing linkage with administrative claims, we were able to study a population with high unmet needs. Several studies have previously demonstrated use of administrative claims to identify CNS and spinal cord metastases with a high degree of sensitivity and specificity.^19^, ^28^ In turn, this information provides important insight for the evaluation of progression‐free survival and cancer relapse in broader populations and enables identification of patients with high unmet needs. Moreover, use of linked SEER‐Medicare database provided a unique opportunity to evaluate patterns of care in a large, well‐defined, geographically and ethnically diverse older US population.

## 5 CONCLUSION

The number of effective therapies for metastatic melanoma has expanded, bringing significant improvements to the control of extracranial disease and marked improvement in survival outcomes, but the burden of disease associated with CNS metastasis in patients with metastatic melanoma remains significant. Patients with CNS metastasis continue to have high unmet medical need, limited treatment options, and poor survival outcomes. Our results confirmed findings from several clinical trials demonstrating improved survival for patients with CNS metastasis treated with BRAF‐targeted therapy or immunotherapy. Initial studies of combination therapies appear promising. Based on these results, further explorations of combinations of radiotherapy, targeted therapy, and immunotherapies are warranted. Continued monitoring of survival and treatment patterns in a contemporary population‐based cohort can provide important information for physicians, patients, and other healthcare professionals by improving understanding of the clinical effectiveness of available treatments and facilitating clinical decision‐making.

## CONFLICTS OF INTEREST

Natalia Sadetsky, Chris J. Wallick, Edward F. McKenna, and Dawn E. Colburn are employees of Genentech/F. Hoffmann‐La Roche and hold stocks/shares in Genentech/F. Hoffmann‐La Roche. Alexandra Hernandez is an employee of Genentech/Hoffmann‐La Roche and a former employee of PlanetPharma. Andy Surinach is an employee of Genesis Research, which receives consulting fees from Genentech/F. Hoffmann‐La Roche for analytic support.

## AUTHOR CONTRIBUTIONS

**Natalia Sadetsky:** Conceptualization, investigation, writing—initial draft, and writing—review and editing. **Alexandra Hernandez:** Conceptualization, investigation, writing—initial draft, and writing—review and editing. **Chris J. Wallick:** Conceptualization, investigation, and writing—review and editing. **Edward F. McKenna:** Conceptualization, investigation, writing—initial draft, and writing—review and editing. **Andy Surinach:** Conceptualization, investigation, writing—initial draft, and writing—review and editing. **Dawn E. Colburn:** Investigation and writing—review and editing.

## Data Availability

Data supporting the findings of this study were used under license from SEER‐Medicare. Restrictions apply to sharing of these data under the terms of the SEER‐Medicare Data Use Agreement and the data are not publicly available.
